# Can Current Innovations in Diagnostic and Therapeutic Strategies Transform Gastroesophageal Cancer Care?

**DOI:** 10.34172/mejdd.3376

**Published:** 2026-01-31

**Authors:** Sakhr Alshwayyat, Noor Almasri, Karam Maraqa, Shatha Abu Samrah, Salsabeel Aljawabrah, Nermeen Abuhalaweh, Yamen Alshwaiyat, Raghad Al-Shami, Layan Al-Zoubi, Yara Alswaiti, Hebah Altaweel, Radwan Abid, Kholoud Alqasem, Mohammed Al-Mahdi Al-kurdi

**Affiliations:** ^1^Kern Medical (UCLA David Geffen School of Medicine affiliate), Bakersfield, California, USA; ^2^Applied Science Research Center, Applied Science Private University, Amman, Jordan; ^3^King Hussein Cancer Center, Amman, Jordan; ^4^Faculty of Medicine, University of Jordan, Amman, Jordan; ^5^Internship, Prince Hamza Hospital, Amman, Jordan; ^6^Internship at Princess Basma Hospital, Irbid, Jordan; ^7^Faculty of Medicine, University of Jordan, Jordan; ^8^University of Jordan, Jordan; ^9^Jordan University of Science and Technology, Irbid, Jordan; ^10^School of Medicine, University of Jordan, Amman, Jordan; ^11^Faculty of Medicine, Hashemite University, Zarqa, Jordan; ^12^Faculty of Medicine, Jordan University of Science and Technology, Irbid, Jordan; ^13^School of Medicine, Jordan University of Science and Technology, Irbid, Jordan; ^14^Department of Internal Medicine, King Hussein Cancer Center, Amman, Jordan; ^15^Faculty of Medicine, University of Aleppo, Aleppo, Syrian Arab Republic

**Keywords:** Biomarkers, Esophageal cancer, Gastric cancer, Global health, Prognosis, Treatment outcome

## Abstract

Gastric and esophageal cancers pose major global health challenges due to high incidence, frequent late-stage diagnosis, and poor survival outcomes. This review synthesized current evidence on emerging technologies and innovative approaches shaping research and practice. We included studies that evaluated diagnostic imaging, endoscopic and surgical treatments, molecular biomarkers, staging, screening, and innovative therapeutic approaches in gastric and esophageal cancers. The review revealed notable demographic and geographic disparities in disease incidence and outcomes. Recent advancements in endoscopic techniques, imaging modalities, and staging systems have enhanced early detection and prognostic stratification. Intriguing findings include significant improvements in survival with HER2-targeted and immune checkpoint inhibitor (ICI) therapies, the ability of liquid biopsy to detect resistance mutations and monitor treatment efficacy in real time, and the use of patient-derived organoids to predict individualized responses. Additionally, artificial intelligence (AI) applications have reduced diagnostic variability, thereby refining therapeutic decision-making. Despite promising innovations, further validation of these emerging technologies is required. Integrating advanced imaging, biomarker-based treatments, and AI-driven tools into clinical practice holds the potential to optimize treatment strategies and improve survival outcomes in gastroesophageal cancers.

## Introduction

 Gastric and esophageal cancers are significant global health challenges due to their high morbidity and mortality rates. Gastric cancer (GC) is the fourth most diagnosed cancer worldwide and the fifth leading cause of cancer-related deaths, while esophageal cancer (EC) ranks as the seventh most common cancer and the sixth leading cause of cancer-related mortality.^1^ In 2022, GC caused 1.1 million new cases and 770,000 deaths, while EC accounted for 511,100 new cases and 445,400 deaths.^2,3^ Despite diagnostic and therapeutic advancements, survival rates remain poor, with a 5-year survival rate of 20% for GC and 10–30% for EC.^4,5^

 Significant limitations persist in managing gastric and esophageal cancers. Delayed diagnosis remains a major challenge, with over 70% of GC cases detected at advanced stages leading to poor survival.^6^ Many barriers were found to delay diagnosis and treatment, including limited screening, insurance issues (65%), geographic constraints (50%), and low health literacy (45%).^7^ Atypical presentations, including Trousseau syndrome in 15–25% of cancer patients, further complicate early recognition.^8^ In treatment, late-stage diagnoses limit surgical options, making chemotherapy, targeted therapy, and immunotherapy the primary interventions. However, therapy resistance remains a major hurdle, with some patients progressing despite the use of immune checkpoint inhibitors (ICIs) or targeted drugs.^9^ The lack of biomarker-driven patient selection further complicates personalized treatment.

 Global disparities in access to advanced therapies exacerbate differences in survival. In low-to middle-income countries, only 16.6% of metastatic patients receive ICIs, and programmed death-ligand 1 (PD-L1) testing is performed in only 42.5% of cases owing to high costs.^10^ Disparities in early diagnosis affect racial and ethnic minorities disproportionately.^11^ Artificial intelligence (AI) is transforming cancer care, enhancing diagnostics, and optimizing treatments.^12^ However, outdated AI models pose risks, underscoring the need for continuous data integration and expert validation.^13^ AI-based cost analysis has reduced the financial burden of immuno-oncology, but ethical and regulatory challenges limit its clinical adoption.^14^ This review evaluated advancements in diagnosis, staging, and treatment, highlighting emerging biomarkers, novel therapies, and AI-driven innovations, while addressing key challenges in cancer care and healthcare disparities.

## Clinical Presentation and Diagnostic Methods

 Gastric and esophageal cancers are often asymptomatic in the early stages and present with non-specific symptoms such as weight loss, anorexia, dysphagia, odynophagia, abdominal pain, nausea, occult bleeding or melena, and anemia.^15^ Dysphagia predicts poor overall survival (OS) in advanced EC ^16^, whereas anemia is a prognostic factor in GC.^17^ Despite these insights, efforts to standardize symptom-based predictive scales have shown limited success.^18^

## Endoscopic and Imaging Techniques

 Advanced endoscopy techniques, such as texture and color enhancement imaging and 3G narrow-band imaging (NBI) enhance lesion detection, particularly for gastric neoplasms.^19,20^ However, endoscopy alone cannot reliably determine subepithelial invasion. Endoscopic ultrasound with fine-needle biopsy (EUS-FNB), improves the diagnostic accuracy for subepithelial lesions.^21,22^ A summary of the relevant diagnostic tools, study types, and outcomes of the various imaging techniques is provided in Table 1.

**Table 1:** Summary of diagnostic tools and outcomes.

**Reference **	**Lesion Layer**	**Diagnostic Tool**	**Study Type**	**Sample Size**	**Conclusion**	**Limitations**
Verloop et al. 2024 ^8^	Subepithelial lesions of the upper GI tract	EUS-FNB, MIAB, EUS-FNA, endoscopic biopsies	Systematic review and meta-analysis	94 studies included	MIAB and EUS-FNB have the highest diagnostic yield (88.2% and 84.2%, respectively)	Different diagnostic yields and adverse event rates based on the technique​
Liu et al. 2024 ^12^	Muscularis propria of the stomach	Precutting EBLR vs. ESD	Randomized controlled trial	40 patients planned for trial	EBLR is less complex and has a lower risk than ESD	Lack of pathological examination in the original EBL technique​
Li et al. 2024 ^13^	Muscularis propria of the stomach	Precutting EBLR	Retrospective cohort study	16 patients	Safe and effective with a 100% R0 resection rate	Risk of delayed perforation without additional snare resection​
Zhang et al. 2024 ^11^	T1b esophageal carcinoma	ESD vs. surgery	Retrospective cohort study	120 patients	Comparable survival outcomes, lower complication rates with ESD	Higher risk of recurrence in certain subgroups​
Kadota et al. 2024 ^7^	Gastric neoplasms	3G-NBI, TXI, WLI	Randomized phase II trial	901 patients	3G-NBI showed higher GN detection rates (7.3%) compared to TXI (5.0%) and WLI (5.6%)	Missed GNs, differences in detection rates between methods​
Futakuchi et al.2023^6^	Gastric neoplasms	TXI (using conventional and new endoscopes)	Prospective Clinical trial	52 gastric neoplasms analyzed	Improved visibility and differentiation of gastric neoplasms with TXI	Dependence on endoscopic technology for effectiveness​

**Abbreviations:** GI: gastrointestinal; EUS-FNB: endoscopic ultrasound-fine needle biopsy; MIAB: mucosal incision-assisted biopsy; EUS-FNA: endoscopic ultrasound-fine needle aspiration; Precutting EBLR: precutting endoscopic band ligation-assisted resection; ESD: endoscopic submucosal dissection; EBL: endoscopic band ligation; 3G-NBI: third generation- narrow band imaging; TXI: texture and color enhancement imaging; WLI: white light imaging; GN: gastric neoplasm.

 Endoscopic mucosal resection (EMR) and endoscopic submucosal dissection (ESD) enable deeper tissue analysis.^23^ ESD carries a risk of perforation, and newer techniques such as precutting endoscopic band ligation-assisted resection (EBLR) aim to mitigate this risk. Precutting EBLR reduces complications and costs while enabling complete resection, although further refinement is needed for larger lesions.^24,25^

 Liquid-based cytology (LBC) and cell block (CB) enhance the diagnostic accuracy for tissue obtained via EUS-FNA, achieving 98.8% accuracy for women and 97.7% for men. Immunohistochemistry adds value to LBC, achieving 81% positive immunostaining, particularly when CB is unavailable or insufficient.^26,27^

 Barium swallow is effective for visualizing the gastrointestinal tract and offers morphological classification, but lacks precision for invasion depth or metastasis assessment. Multi-slice spiral CT has comparable diagnostic accuracy.^28^ Diagnostic laparoscopy is crucial for staging but may increase the peritoneal seeding risk, limiting its use in patients with a high risk of peritoneal metastasis (Stage IIIc).^29^

 Beyond diagnosis, many tools are used to assess lesions preoperatively, aiding in the determination of the most effective therapies. The utility of various tools for preoperative assessment of gastric and esophageal cancer lesions was evaluated (Table 2).

**Table 2:** Preoperative assessment tools and their clinical utility

**Preoperative assessment tool**	**Study type**	**Indication **	**Evidence **
MRI^18^	Meta-analysis	to predict the response to adjuvant chemotherapy and radiotherapy in esophageal cancer	DWI > DCE Sensitivity: 0.82 Specificity: 0.81
DECT	Prospective	predict serosal invasion of gastric cancer preoperatively, stratify patients into high and low-risk groups ^19,20^	HR: 2.233 (training cohort) HR: 3.042 (validation cohort 1)^20^
	retrospective	Evaluate occult peritoneal metastasis	PPV: 0.75 ^21^
ECV	Retrospective	Predict pathological complete regression post preoperative immune chemotherapy in advanced GC	odds ratio: 0.911 ^22^
PET tracers	Cohort study	Radiotherapy planning	minor change: 16% major change: 9% ^23^
	Retrospective	Compare single to dual tracers	GTV mean difference: 31.8 FTV total (Pearson’s correlation): *P* < 0.001 ^24^
Multimodality	Prospective	Assessment of pathological regression outcomes of gastric cancer using spectral CT and MRI	HR: 2.508 ^25^

## Biomarkers

 Advances in biomarkers and the tumor microenvironment have revolutionized oncology by enabling targeted therapies, reducing the adverse effects of non-specific treatments, and predicting immunotherapy responses and tumor behavior.^30^ A summary of the key biomarkers and their clinical implications is provided in Table 3.

**Table 3:** Biomarkers and their clinical relevance in esophageal and gastric cancers

**Reference**	**Biomarker**	**Targeted Population**	**Study Type**	**Objectives**	**Sample Size**	**What’s New?**	**Next Step**
Gao et al. 2024 ^38^	Angiogenesis-related proteins (IL-8, TIE2, HGF)	Esophageal cancer patients	Cross sectional	Evaluate serum proteins as biomarkers for immunotherapy response	91	Identified three key proteins associated with survival outcomes in immunotherapy	Verification in larger, prospective studies
Li et al. 2024 ^35^	NKG2A and PD-1	Gastric cancer patients	Retrospective Cohort Study	Analyze immune checkpoint expression for treatment resistance	Data not specified	Identified patterns of immune checkpoint expression affecting anti-PD-1 resistance	Further exploration of combination therapies
Zúñiga-Pérez et al. 2024 ^29^	CSTB, TPI1, DMBT1	Patients at risk of gastric cancer	Systematic Review	Assess the diagnostic accuracy of salivary biomarkers	Data not specified	Highlighted three highly accurate proteins for early detection	Enhancement of study designs and validation of biomarkers
Shitara et al. 2024 ^34^	HER2-associated biomarkers	HER2-positive gastric cancer patients	Phase 2 Clinical Trial	Evaluate the efficacy of Trastuzumab deruxtecan	Primary cohort size not specified	Correlated HER2 biomarkers with therapy response	Larger studies to explore resistance mechanisms
Chen et al. 2024 ^32^	PD-L1 expression positivity scores	Advanced esophageal squamous cell carcinoma patients	Network Meta-Analysis	Compare immunotherapy combinations	4688	Highlighted the value of PD-L1 scores in predicting treatment efficacy	Further clinical trials and refinement of therapy combinations
Wang et al. 2024 ^31^	CLDN18-ARHGAP fusion gene	Gastric cancer patients with the fusion gene	Retrospective Cohort Study	Investigate the therapeutic potential of targeting CLDN18-ARHGAP	87	Showed robust anti-tumor capacity and immune suppression involvement	Clinical trials for PI3K inhibitors and further molecular studies
Waters et al. 2024 ^37^	Claudin 18 Isoform 2 (CLDN18.2)	Patients with gastric and gastroesophageal junction adenocarcinoma	Retrospective Cohort Study	Assess the prevalence and prognostic value of CLDN18.2	304	Found significant associations with clinical outcomes and tumor characteristics	Larger, prospective studies to validate findings
Qi et al. 2024 ^33^	HIF1A-related genes	Gastric cancer patients	Observational Study (mouse model)	Explore HIF1A's role in immune evasion and therapy resistance	Data not specified	Identified key mechanisms of immune evasion influenced by HIF1A	Development of therapies targeting these pathways
Zhang et al. 2024 ^30^	CLDN18-ARHGAP26	Patients with diffuse gastric cancer	Observational Study (mouse model)	Determine the oncogenic role of CLDN18-ARHGAP26 fusion	Data not specified	Demonstrated oncogenic activity and identified potential therapeutic targets	Evaluation of FAK and YAP-TEAD inhibitors in clinical trials
Santos et al. 2024 ^28^	EBV and MSI-H	Gastric adenocarcinoma patients	Meta-Analysis	Relate molecular subtypes with epidemiological factors	25 studies included	Showed sex-specific prevalence of EBV + and MSI-H subtypes	Further research on subtype-specific therapies
Cheng et al. 2024 ^36^	HER2	Gastric cancer patients	Meta-Analysis	Assess the prognostic significance of HER2 expression in gastric cancer	9945	Demonstrated HER2 overexpression as a significant indicator of poor prognosis in both resectable and advanced gastric cancer	Encourage further evaluation of HER2 as a therapeutic target in resectable gastric cancer

 Microsatellite Instability (MSI-H) is more prevalent in women with gastric adenocarcinoma, suggesting potential sex-based differences in the tumor microenvironment and raising the possibility of gender-specific therapies [30]. Salivary biomarkers have also shown high accuracy in the early diagnosis of gGC.^31^

 GC, which is often resistant to several drugs, highlights the need for new therapeutic targets. The CLDN18-ARHGAP26 fusion gene activates pathways such as FAK and YAP/TEAD, the inhibition of which produces robust antitumor effects in vitro and in vivo, especially in diffuse GC.^32^ Additionally, the CLDN18-ARHGAP fusion gene generates immunogenic neoantigens that activate PI3K/AKT-mTOR-FAS signaling, offering a promising therapeutic target.^33^ PD-L1 positivity in gastric and esophageal cancers correlates with better OS and an improved response to ICIs, regardless of expression levels.^34^

 High recurrence rates in GC have prompted investigations into tumor microenvironment heterogeneity. Quiescent cancer cells (QCCs) with high HIF1A expression levels are resistant to therapy. Silencing HIF1A in mouse models increases T-cell sensitivity and apoptosis rates when combined with anti-PD1 therapy.^35^ FGFR2 gene amplification associated with anti-HER2 resistance showed a 0% objective response rate (95% CI 0–45.9%) in trials, although further validation is needed.^36^ Co-expression of PD-1 and NKG2A inhibits CD8 + T cell proliferation, with NKG2A emerging as a novel target to overcome anti-PD-1 resistance.^37^ Moreover, biomarkers can predict cancer prognosis. HER2 positivity in GC indicates worse prognosis (HR 1.37).^38^ CLDN18.2 positivity in gastric and gastroesophageal junction (GEJ) adenocarcinoma shows a 73% concordance rate between primary and metastatic tumor samples.^39^ Angiogenesis-related proteins (IL-8, TIE2, and HGF) predict the prognosis and treatment response in EC.^40^

## Staging

 Survival rates in esophagogastric cancers vary significantly according to the stage. European patients with early-stage disease (Stage I) have a 67-68% 5-year survival rate compared with 19-47% for Stage II-III (locally advanced) and 2-3% for Stage IV (metastatic disease).^41^ Accurate staging is essential for treatment planning and prognosis.^42^ Advancements in imaging modalities have significantly enhanced the accuracy of the clinical TNM staging. EUS is highly effective for T staging, demonstrating pooled sensitivities of 81.6% for T1, 81.4% for T2, 91.4% for T3, and 92.4% for T4 stages, with specificities ranging from 96.3% to 99.4%. For gastric adenocarcinoma, EUS showed an overall accuracy of 52.8%, performing best for T1 cancers with 85.23% accuracy. Despite its utility, EUS accuracy decreases in more advanced stages, necessitating complementary imaging techniques.^43,44^

 Computed tomography (CT) remains one of the most commonly used modalities for preoperative evaluation, but it is less effective in detecting early-stage tumors owing to poor soft-tissue resolution.

 However, combining preoperative CT features with TNM staging and tumor differentiation enhanced its diagnostic power, achieving an AUC of 0.819, with sensitivity and specificity of 66.7% and 83.8%, respectively. Despite its efficacy, CT has limitations in patients with ionizing radiation exposure.^45,46^

 Magnetic resonance imaging (MRI) offers excellent soft-tissue resolution without radiation exposure and outperforms EUS in T3/T4 staging for EC with higher specificity (93% vs. 59%) and accuracy (96% vs. 81%). Additionally, MRI with orthogonal axial imaging surpasses standard MRI and CT for GC staging, achieving accuracy rates of 88.7-94.7%, sensitivity of 66.7-93.0%, and specificity of 91.5-100%.^46^

## Treatment

###  Surgical Treatment in Gastric and Esophageal Cancer

 Surgery remains the primary curative option for resectable esophageal and gastric cancers, with outcomes varying according to patient factors, tumor stage, location, and size. However, conventional open surgeries, particularly for complex tumor locations such as esophagectomy, are challenging because of the multiple incision sites in the neck, thorax, abdomen, and transhiatal approach.^47^ Advances in minimally invasive and robot-assisted techniques have improved these results.

 Tumor sites significantly affect surgical outcomes. Proximal gastrectomy (PG) has been extensively studied for tumors in the upper third of the stomach and has shown significant benefits in reducing post-gastrectomy complications, including anemia, dumping syndrome, gastroesophageal reflux, dietary dissatisfaction, and weight loss. It presents a better alternative to total gastrectomy in such cases.^48,49^

 Laparoscopic gastrectomy and esophagectomy demonstrate superior outcomes compared with open surgery, which is attributed to smaller incisions, faster recovery, and improved postoperative nutritional status, particularly in patients with locally advanced GC (LAGC) patients receiving neoadjuvant chemotherapy. They also reduce wound infections and hospital stay.^50,51,52^ Beyond laparoscopy, robot-assisted surgery yields favourable results. Robotic gastrectomy has shown promise in reducing morbidity while achieving outcomes comparable to or better than laparoscopic gastrectomy,^53,54^ with shorter hospital stays, lower blood loss, and an easier learning curve. Similarly, robot-assisted esophagectomy (RAMIE) has gained popularity for addressing challenges such as intrathoracic anastomosis during esophagectomy. A study of 5,170 patients found that RAMIE improved survival, with a median survival of 42 months, compared with higher mortality risks with open esophagectomy (HR = 1.19) and laparoscopic esophagectomy (HR = 1.22).^55,5,6^

###  Endoscopic Therapies

 Endoscopic therapies are a crucial curative option for early-stage gastric and esophageal cancers.^57,58^ These include EMR and endoscopic submucosal dissection (ESD). Lei and others (2024) reported that endoscopic treatments had the least impact on quality of life (QoL) over 5 years, with only a 7.5% decline, compared with 37.8% for chemoradiotherapy over 2 years and 12.2% for esophagectomy after 1 year.^59^ EMR is effective for small lesions ( < 2 cm) with minimal complications, although its success declines for larger lesions or those with ulcers or submucosal fibrosis.^60^ ESD, which is better suited to larger lesions, involves lesion identification, submucosal saline injection, and dissection. Zhang and co-workers demonstrated improved outcomes when ESD was combined with teprenone.^61^

###  Chemotherapy and Chemoradiation

 Systemic treatments, including chemotherapy and chemoradiation, are widely used for esophageal and gastric cancer but often cause significant complications, such as chemotherapy-induced peripheral neuropathy, negatively impacting QoL.^62^ Perioperative chemotherapy is the standard treatment for stage IB-III gastric cancer. Trials such as INT0116, CLASSIC, and FLOT4-AIO have demonstrated survival benefits.^63^ A Brazilian study reported a 3-year OS rate of 79.8% with the CLASSIC regimen, although only 56% of patients completed treatment due to intolerance.^64^ However, an international trial found no survival benefit of adding preoperative chemoradiotherapy to perioperative chemotherapy for resectable gastric and GEJ adenocarcinoma.^65^

 Chemotherapy is the first-line treatment for advanced EC.^66^ Neoadjuvant chemoradiation or chemotherapy is often recommended for non-metastatic cases, with benefits primarily for patients achieving a pathological complete response (PCR).^67^ A retrospective study of 57,116 patients with stage II/III EC showed that perioperative chemotherapy and tri-modality therapy significantly improved OS compared with definitive chemoradiation. The median survival was 5.5 years for perioperative chemotherapy vs. 1.5 years for definitive chemoradiation.^68^

## Biomarker-Oriented Treatment Strategies

 Although chemotherapies target proliferating cells with significant side effects, advancements in drugs that specifically target antigens and activate the immune system to destroy cancer cells have revolutionized the treatment of advanced gastric and esophageal cancers. Given the heterogeneity of these tumors, targetable antigens such as HER2 and PD-L1 form the basis of biomarker-oriented immunotherapy, showing promising efficacy depending on the histopathology and antigen expression levels.^69,70^ Figure 1 highlights key biomarkers and emerging therapies in gastroesophageal cancer treatment.

**Figure 1:**
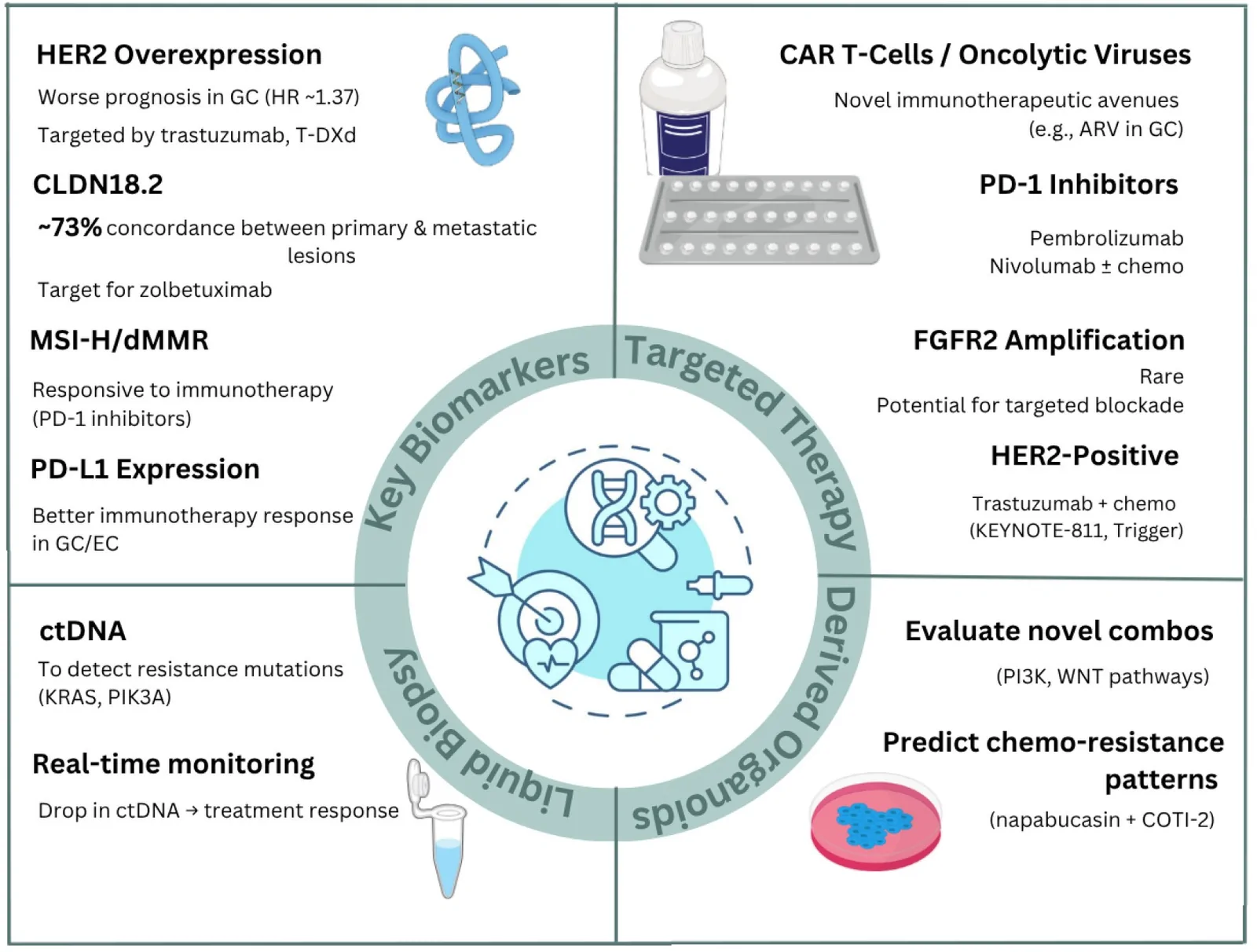
Key molecular biomarkers and emerging treatment approaches for gastroesophageal cancer

## Biomarker-Oriented Treatment Strategies: HER2-positive Esophageal and Gastric Adenocarcinoma

 The combination of trastuzumab (a monoclonal anti-HER2 antibody) and chemotherapy remains the standard treatment for HER2-positive esophageal and gastric adenocarcinomas. The phase 3 KEYNOTE-811 trial assessed the addition of pembrolizumab to standard therapy in 350 patients with HER2-positive metastatic GEC, compared with 346 patients without pembrolizumab. Pembrolizumab improved progression-free survival (PFS) and OS, particularly in patients with high PD-L1 expression.^71^

 The phase II CAPOX BETR trial evaluated standard capecitabine and oxaliplatin therapy with or without trastuzumab in HER2-positive GEC. Bevacizumab (a VEGF-A inhibitor) was administered to assess its potential benefits. Despite a median PFS of 14 months, the study’s small sample size (n = 36) and demographic bias (young white men) limited its applicability.^72^ A different phase II trial explored HLX22 (an anti-HER2 antibody) with a trastuzumab biosimilar (HLX02) and XELOX chemotherapy in HER2-positive GC. High-dose HLX22 patients had superior PFS (15.1 months) compared with placebo (8.2 months), suggesting its potential to improve therapy, although limited patient numbers limit study validity.^73^

## Biomarker-Oriented Treatment Strategies: HER2-negative Esophageal and Gastric Adenocarcinoma

 HER2-negative adenocarcinoma is classified by mismatch repair (MMR) status as deficient (dMMR) or proficient (pMMR). The ARMANI study (2024) evaluated 280 patients who received paclitaxel with ramucirumab or continued oxaliplatin-based chemotherapy (FOLFOX or CAPOX). The paclitaxel-ramucirumab group had a PFS of 8.8 months, compared with 6.1 months in the control group, suggesting its potential as a promising alternative for advanced HER2-negative gastric or GEJ cancer. ^74^ For dMMR or MSI-H tumors, ICIs are recommended as first-line monotherapy, with combined ICIs and chemotherapy used as second-line therapies.^75^

 In pMMR cases, monoclonal antibodies targeting specific antigens are promising. The randomized phase 3 ATTRACTION-4 trial found that nivolumab plus chemotherapy achieved 80% three-year OS, demonstrating long-term efficacy.^76^ A randomized phase 3 trial involving 1,254 patients with locally advanced GC or GEJ adenocarcinoma compared pembrolizumab plus cisplatin-based chemotherapy with chemotherapy alone. Although pembrolizumab improved event-free survival, the difference was not statistically significant.^77^ A phase 2 trial of sintilimab found that 79.3% of patients experienced tumor downstaging.^78^

 The COMPASSION-04 trial met its primary endpoint with an overall response rate (ORR) of 52.1% and a median OS of 17.64 months in patients with PD-L1 CPS < 1. This trial evaluated cadonilimab, a bispecific antibody targeting PD-1, in combination with chemotherapy.^79^ Similarly, the PLATFORM study examined durvalumab, an anti-PD-L1 antibody, as a maintenance therapy for HER2-negative advanced esophagogastric adenocarcinoma. No significant PFS difference was observed between the durvalumab and surveillance groups, indicating a limited benefit.^80^

 Beyond anti-PD-1 antibodies, targeted therapies include fruquintinib, a selective tyrosine kinase inhibitor (VEGFR). The phase 3 FRUTIGA trial evaluated fruquintinib plus paclitaxel in 703 patients and successfully met the primary PFS endpoint.^81^ The SPOTLIGHT and GLOW trials assessed zolbetuximab, a monoclonal antibody targeting claudin-18 isoform 2, in gastric/GEJ adenocarcinomas. PFS improved slightly compared with placebo (12.94 vs. 12.65 months), warranting further investigation.^82^ A summary of key trials evaluating treatment strategies for both HER2-positive and HER2-negative esophageal and gastric adenocarcinomas is provided in

## Biomarker-Oriented Treatment Strategies: Esophageal Squamous Cell Carcinoma (SCC)

 Patients with SCC are stratified by PD-L1 CPS levels.

- CPS ≥ 1: First-line treatment includes immunotherapy plus chemotherapy.
- CPS < 1: Chemotherapy alone is preferred.

A novel chemotherapy-free regimen combining surufatinib and toripalimab was evaluated in an open-label study targeting GC, GEJ adenocarcinoma, and esophageal SCC. The results showed encouraging antitumor effects and safety in immunotherapy-naïve patients, warranting larger trials.^83^ The effectiveness of immunotherapy underscores the need for large-scale trials to optimize outcomes and assess immune-mediated side effects comparable to chemotherapy-induced toxicities.^34^ Emerging combination immunotherapy protocols require further refinement to balance their efficacy and safety.^34^

## New Emerging Therapies

 The limited success of conventional treatments has driven innovation toward more targeted therapies. IgG-triggering antibody treatments and oncolytic viruses represent novel strategies that harness the immune system to target tumor cells with greater specificity and reduced toxicity.

 A peptide-based vaccine, HER-Vaxx, designed for HER2-positive GC, was evaluated in a randomized phase II study (n = 19).^84^ It reported a 40% OS benefit, with a median OS of 13.9 months in patients receiving HER-Vaxx plus chemotherapy, compared to 8.31 months with chemotherapy alone. HER2-specific antibodies generated by the vaccine enhance tumor reduction and improve chemotherapy efficacy.^84^ In addition to cancer vaccines, oncolytic viruses, such as avian reovirus (ARV), have shown potential in GC by inducing TRAIL expression in peripheral blood mononuclear cells (PBMCs), leading to apoptosis and immune activation.^85^ These findings support preclinical development of ARV as an adjunct therapy for GC (Table 4).

**Table 4:** Key trials evaluating treatment strategies for both HER2-positive and HER2-negative esophageal and gastric adenocarcinomas

**Reference**	**Study Type**	**Sample Size**	**Population**	**Objective**	**Key Findings**	**Future Recommendation**
Gao et al. 2024 ^20^	Phase 1b/2 Clinical Trial	98 patients	Patients with HER2-negative unresectable advanced or metastatic gastric or gastroesophageal junction (GEJ) adenocarcinoma	Evaluate the efficacy and safety of cadonilimab plus chemotherapy as first-line treatment	The regimen showed a 52.1% objective response rate, with a median progression-free survival of 8.18 months and overall survival of 17.48 months.	Further optimization of drug combinations and doses to improve therapeutic effects while mitigating toxicities.
Wang et al. 2024 ^21^	Phase 3 Randomized Clinical Trial	703 patients	Patients with advanced gastric or gastroesophageal junction adenocarcinoma who progressed on fluorouracil- and platinum-containing chemotherapy	Compare efficacy and safety of fruquintinib plus paclitaxel versus placebo plus paclitaxel as second-line treatment	Progression-free survival was significantly improved (5.6 vs. 2.7 months), but overall survival was not significantly different.	Further research needed to explore combination strategies that enhance overall survival outcomes.
Randon et al. 2024^22^	Phase 3 Randomized Clinical Trial	280 patients	Patients with advanced HER2-negative gastric or gastroesophageal junction cancer with disease control after first-line chemotherapy	Assess whether switch maintenance with paclitaxel plus ramucirumab improves outcomes compared to continuation of oxaliplatin-based chemotherapy	Progression-free survival improved (6.6 vs. 3.5 months) with switch maintenance, but a higher incidence of treatment-related adverse events was observed.	Evaluate patient selection criteria to balance benefits and risks of switch maintenance therapy.
Janjigian et al. 2023 ^23^	Phase 3 Randomized Placebo-Controlled Trial	698 patients	Patients with HER2-positive metastatic gastric or gastroesophageal junction adenocarcinoma	Assess the efficacy and safety of pembrolizumab added to trastuzumab and chemotherapy	Significant improvement in progression-free survival (10.0 vs. 8.1 months), but overall survival results did not yet reach statistical significance.	Continue follow-up to determine long-term survival benefits and optimal patient selection for pembrolizumab use.
Li et al. 2024 ^24^	Phase 2 Randomized Clinical Trial	53 patients	Patients with HER2-positive advanced gastric cancer	Evaluate the efficacy and safety of HLX22 plus trastuzumab biosimilar HLX02 and XELOX as first-line therapy	Progression-free survival improved significantly with HLX22 addition (15.1 vs. 8.2 months), with a manageable safety profile.	Further validation in larger phase 3 trials to confirm clinical benefits and long-term safety.

 Natural immunomodulators have also shown promise. A phase IB study investigated β-glucan with camrelizumab (anti-PD-1) and SOX chemotherapy for advanced gastric adenocarcinoma, achieving a median PFS of 10.4 months, suggesting a synergy between immunomodulators and standard treatments.^86^ A multicenter phase II trial compared neoadjuvant camrelizumab, apatinib (an angiogenesis inhibitor), and chemotherapy with chemotherapy alone in locally advanced GC. The combination therapy group achieved a higher major pathological response rate, reinforcing the role of integrated immunotherapy and targeted therapies in neoadjuvant settings.^87^

## Patient-Derived Organoids

 Patient-derived organoids (PDOs) are revolutionizing personalized medicine and accurately replicating tumor phenotypes and molecular features.^88^ A co-clinical trial using PDOs to evaluate FLOT therapy achieved high diagnostic accuracy (AUC = 0.994), differentiating responders from non-responders.^89^ The PDO response also correlated with clinical outcomes and was validated in patient-derived xenograft (PDOX) mouse models.

 A total of 91.7% of patients with GC exhibit treatment responses consistent with their PDO-derived models, underscoring PDOs as predictive tools for therapy selection.^90^ In drug screening, PDOs identified synergistic effects of napabucasin and COTI-2, inhibiting the JAK2/STAT3 and AMPK pathways in GC.^91^ These findings highlight the role of PDOs in personalized medicine, as summarized in Table 5.

**Table 5:** PDO applications in personalized medicine

**Reference**	**PDO Application**	**Key Findings**	**Therapeutic Impact**
Schmäche et al. 2024 ^89^	Organoids for predicting chemotherapy responses	Established a predictive threshold for FLOT regimen response	Personalized treatment plans for better outcomes
Zhao et al. 2024 ^90^	Biobank for personalized drug screening	Organoids reproduce patient-specific chemo responses	Prediction of individual responses to chemotherapy
Xu et al. 2024 ^91^	Organoids for drug sensitivity testing	Maintained original tumor characteristics; varied drug sensitivities	Personalized medicine development
Jiang et al. 2024 ^1^	Review of success rates in organoid culture	Identified factors influencing culture success rates	Optimization of organoid culture techniques
Ota et al. 2024 ^92^	Coculture model for immune cell interaction with cancer organoids	Enabled real-time immune response observation	Potential prediction of immune checkpoint inhibitor effects
Kim et al. 2024 ^93^	Vascularized organoid model for drug response prediction	Accurate reproduction of patient responses to VEGFR2 inhibitors	Personalized drug testing and discovery
Sharpe et al. 2024 ^94^	Assembloids for mimicking tumor microenvironment	Faithfully replicated tumor differentiation and fibroblast phenotypes	Enhanced understanding of cancer-stroma interactions

 PDOs predict chemoresistance. A study of 57 PDOs from 73 GC patients found that chemoresistant PDOs upregulated PI3K and WNT signalling, while chemosensitive PDOs upregulated tumor suppressors, highlighting distinct genetic profiles.^90^

 Advancements in PDO co-culture models include the development of immune cell-organoid co-culture models by Hiroshi and others, which enable the real-time monitoring of immune responses to checkpoint inhibitors (Table 6).^92^ In addition, vascularized organoid models (VOMs) have predicted responses to VEGFR2-targeted therapy in GC.^93^ Furthermore, combining PDOs with cancer-associated fibroblasts (CAFs) has clarified the tumor-stromal interactions that influence resistance.^94^ Recent developments in PDO co-culture models are summarized in **q**.

**Table 6:** Organoid co-culture models

**Reference**	**Type of Model**	**Components**	**Mechanism **	**Applications**	**Limitations**
Kim et al. 2024 ^6^	Vascularized organoid model (VOM)	Gastric cancer organoids, perfusable endothelium, stomach decellularized ECM	Reproduces the molecular-pathologic features of tumors, allowing for accurate prediction of drug response.	Personalized medicine and drug discovery for gastric cancer.	Limited by the current understanding of the complete tumor microenvironment; may not fully replicate in vivo conditions.
Sharpe et al. 2024 ^7^	Assembloid models with CAFs and organoids	Esophageal adenocarcinoma organoids, cancer-associated fibroblasts (CAFs)	Mimics tumor-stroma interactions and provides insights into therapy resistance and disease progression.	Study of cancer-stroma interactions and testing new therapeutic strategies.	May not completely capture the full complexity of the tumor microenvironment, particularly interactions with immune cells and other stroma components beyond fibroblasts.
Ota et al. 2024 ^5^	3D coculture model	Gastric cancer organoids, immune cells	Allows immune cells to move freely and maintain contact with cancer organoids for real-time observations.	Predicting effects of immune checkpoint inhibitors before clinical use.	Model complexity might limit straightforward interpretation of immune response due to multiple interacting components.

## Liquid Biopsy and Circulating Tumor DNA (ctDNA)

 Compared with traditional biopsies, liquid biopsy is a less invasive method that analyses circulating tumor DNA (ctDNA) and circulating tumor cells (CTCs) to monitor treatment response and detect recurrence.^95,96^ Table 7 summarizes key applications of liquid biopsy in GEC.

**Table 7:** Applications of liquid biopsy in gastroesophageal cancer

**Reference**	**Liquid Biopsy Biomarker**	**Application**	**Key Findings**	**Clinical Implications/Outcome**
Li et al. 2024 ^12^	cfDNA kinetics	Prognosis and radiotherapy effect in esophageal cancer	Pre-radiotherapy cfDNA levels correlate with clinical stage and are linked to OS and PFS; kinetics post-RT can also predict treatment effectiveness.	Offers a potential clinical application for non-invasively monitoring and predicting the outcomes of radiotherapy in esophageal cancer.
Tatalovic et al. 2024 ^13^	ctDNA	Early response monitoring in gastroesophageal cancer	ctDNA dynamics within two weeks of treatment start can predict therapy response, linked with OS and PFS.	Rapid assessment of treatment efficacy, potentially leading to adjustments in therapy plans much earlier than current methods allow.
Nixon et al. 2024 ^14^	ctDNA	Therapy response in pembrolizumab-treated gastroesophageal cancer	Ultra-sensitive ctDNA assay captures dynamics correlating with therapy response, predicting improved patient outcomes.	Enhances treatment decision-making by providing early indications of therapy success or failure, which could lead to personalized treatment adjustments.
Iden et al. 2024 ^15^	ctDNA	Predicting recurrence and survival in gastric and GEJ cancer	ctDNA after surgery correlates with recurrence risk and survival, providing a non-invasive prognostic tool.	The presence of ctDNA post-surgery can guide more aggressive or altered postoperative treatment strategies to improve survival rates.
Richter et al. 2023 ^16^	ctDNA	Monitoring therapy in esophageal cancer	ctDNA levels correlate with therapy response, helping in monitoring treatment efficacy.	Combining ctDNA with solid biopsies can provide comprehensive insights into tumor dynamics, assisting in the precise tailoring of treatment regimens.
Jung et al. 2023 ^17^	ctDNA	Monitoring systemic chemotherapy for HER2 + gastric cancer	ctDNA profiles useful for predicting treatment response.	Early ctDNA changes can predict progression before it appears on CT, improving treatment personalization and potentially enhancing outcomes.
Zhang et al. 2024 ^18^	ctDNA	Prognostic evaluation in esophageal cancer	Positive correlation between ctDNA presence and poor prognosis in terms of OS, DFS/RFS, and PFS.	Plasma ctDNA serves as a robust prognostic marker for esophageal cancer, indicating worse outcomes.
Ding et al. 2024 ^19^	mRNA biomarkers	Early detection of recurrence in gastric cancer	Four mRNA biomarkers linked to recurrence, identified through transcriptomics, show correlation with reduced recurrence and metastasis.	A non-invasive approach that enhances recurrence detection, improving patient management and personalized treatment strategies.

## Role of ctDNA in Monitoring Treatment Efficacy

 ctDNAs enable rapid treatment monitoring. In metastatic GEC (mGEC), Tatalovic and colleagues found that a 57.1% decrease in ctDNA within two weeks of chemotherapy correlated with improved OS and PFS, allowing clinicians to predict treatment efficacy up to 80% faster than CT scans.^95^ Jung and others evaluated serial ctDNA in 15 HER2-positive metastatic GC patients undergoing systemic chemotherapy and ICIs and detected tumor progression 2–24 weeks before CT imaging.^97^ Another cohort study (n = 25) found that ctDNA detected progression 2 months earlier than imaging.^98^

 A study of 86 patients with resectable GC or GEJ cancer found that baseline ctDNA levels decreased from 56% to 37% after one chemotherapy cycle, then to 25% preoperatively and 15% post-surgery, demonstrating its role in monitoring treatment response.^99^ ctDNAs also play a role in therapeutic optimization by identifying resistance mutations. In patients with HER2-positive GC, 93% have baseline ctDNA mutations, including KRAS and PIK3A mutations.^97^

## Prognostic Significance of ctDNA in Recurrence Detection and Therapy Optimization

 Persistent ctDNA after chemotherapy is associated with shorter recurrence-free survival (RFS) (HR = 2.54) and OS (HR = 2.23) in patients with GC and GEJ cancer.^99^ A meta-analysis (13 studies, n = 604) found that high ctDNA levels correlated with poor OS, disease-free survival (DFS), and PFS in EC.^96^ Similarly, a retrospective study (n = 88, EC) found that higher pre-radiotherapy ctDNA levels were negatively associated with OS (*P* < 0.001) and PFS (*P* < 0.002), emphasizing the role of ctDNA in risk-adapted surveillance.^100^

 Ding et al. developed a liquid biopsy panel with four mRNA biomarkers, achieving a high predictive accuracy (AUC = 0.919) for tumor recurrence in validation cohorts. Integrating molecular and clinical data enhances risk stratification for personalized oncology.^101^ Real-time genetic monitoring can personalize treatment, reduce therapy failure, and improve outcomes. Incorporating ctDNA into standard practice represents a paradigm shift in cancer management, enabling early detection, real-time monitoring, recurrence prediction, and therapy optimization in precision oncology.

## The Role of Microbiota in the Treatment and Outcome of GEC

 Gut microbiota significantly influences cancer treatment responses and prognosis.

- A cohort study (n = 117, HER2-negative GC) found that higher lactobacillus diversity was correlated with better PD-1/PD-L1 immunotherapy responses and PFS (*P* = 0.057). Findings were validated in a second cohort (n = 101).^102^
- Fusobacterium nucleatum was prevalent in ESCC and linked to worse prognosis and chemoresistance (*P* = 0.017).^103^
- A prospective study (n = 31, ESCC) identified B. plebeius (AUC = 0.865) and B. ovatus (AUC = 0.790) as markers of TP chemotherapy efficacy.^104^
- In a study investigating intratumoral microbiota and response to neoadjuvant chemoimmunotherapy (NACI) in ESCC, high intratumoral Streptococcus abundance predicted better disease-free survival (DFS) (*P* = 0.0341).^105^
- Increased levels of Prevotella salivae and Veillonellaceae in the salivary microbiome correlated with significantly improved chemoradiotherapy response in locally advanced ESCC.^106^
- Butyricimonas and Actinomyces were identified as predictors of postoperative recurrence in EC using machine learning models, achieving an AUC of 0.93.^107^

Microbiota-based interventions may optimize treatment efficacy and advance precision oncology in GEC treatment. Table 8 provides an overview of key microbiota influences on therapy response and clinical outcomes in GEC.

**Table 8:** Microbiota influence on therapy response and outcome

**References**	**Microbial Species **	**Assessment Tool**	**Predictive Outcome**	**Clinical Relevance**
He et al. 2024 ^8^	Prevotella_salivae, Saccharibacteria_TM7_G3_bacterium_HMT_351	16S ribosomal RNA sequencing, Liquid chromatography-mass spectrometry	Association with pathological complete response in chemoradiotherapy	Salivary microbiome variations correlate with host immune response and chemoradiotherapy outcomes in esophageal squamous cell carcinoma.
Otsuka et al. 2024 ^9^	Butyricimonas, Actinomyces	16S rRNA metagenome sequencing, Machine learning (Random forest model)	Predictive marker for postoperative recurrence of esophageal cancer	Intestinal microbiome profiles could serve as non-invasive biomarkers for cancer recurrence prediction.
Guan et al. 2023 ^10^	Various microbiomes associated with different cancer types	Weighted gene coexpression network analysis (WGCNA), Machine learning	Tumor immunity and prognosis across various cancers	Microbiome-genome interactions play significant roles in tumor progression and could inform personalized cancer treatment strategies.
Yang et al. 2023 ^11^	Helicobacter, Halomonas, Shewanella	16S rRNA gene sequencing	Diversity and composition linked to gastric cancer prognosis	Microbiota signatures can serve as biomarkers for clinical outcomes in gastric cancer patients.

## Quality of Life, Survivorship, and the Global Burden of Gastric & Esophageal Cancers

 These cancers significantly affect patients’ QoL, as they present with debilitating symptoms such as dysphagia, unintentional weight loss, and malnutrition in later stages.^108,109^ Managing these symptoms alongside psychological challenges is essential for improving daily functioning and survival outcomes.^110^ Figure 2 illustrates the various challenges and impacts on the QoL and survivorship in patients with GEC, emphasizing the complexity of care required to address these issues.To assess health-related QoL (HRQoL), tools such as the EORTC QLQ-C30 and QLQ-OG25 measure physical, psychological, and social functions, along with symptom severity and financial difficulties, providing a comprehensive evaluation tailored to GEC.^109^

**Figure 2:**
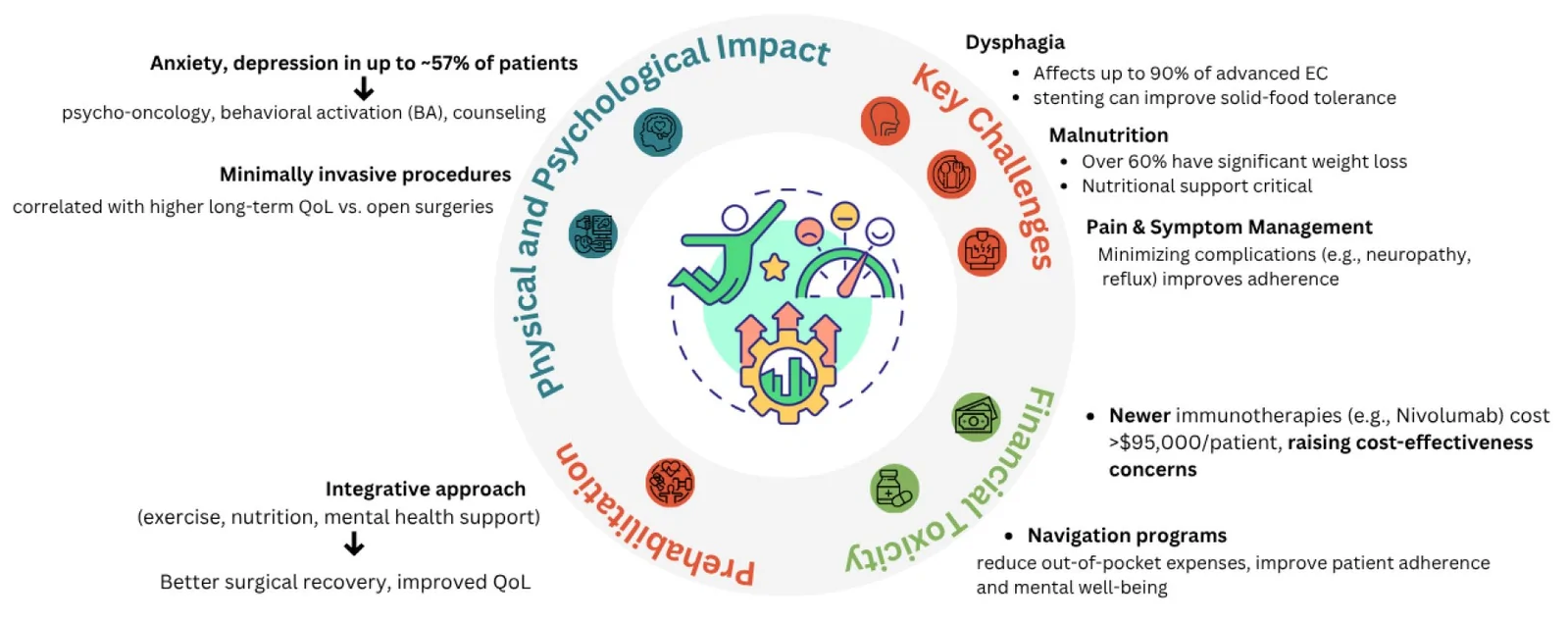
Factors affecting quality of live and survival in gastroesophageal cancer

## Physical and Functional Impact

 While cancer treatments often reduce QoL due to long-term complications, minimally invasive approaches provide better functional outcomes for patients with EC and GC. However, post-surgical QoL often remains suboptimal during the first year owing to anatomical changes and surgical reconstruction techniques.Dysphagia, affecting up to 90% of patients with advanced EC, is among the most distressing symptoms and severely impairs QoL.^111^ Treatments such as self-expandable metal stents (SEMS) offer effective palliation, enabling 90% of patients to tolerate solid foods by the third month post-stenting, while minimizing complications compared with chemo-radiotherapy or photodynamic therapy.^111^ ICIs combined with chemotherapy also improve patient-reported outcomes (PROs) by alleviating symptoms such as dysphagia and pain, while reducing treatment-related side effects such as nausea and vomiting.^112^

 Malnutrition, prevalent in over 60% of patients with esophageal and gastric cancer, exacerbates treatment intolerance, increases complications, and diminishes QoL.^113,114^ Effective management with oral, enteral, or parenteral nutrition can mitigate these effects. Personalized dietary counselling after surgery has shown significant benefits, including reduced BMI loss, improved protein intake, and enhanced QoL 90 days post-surgery in patients with GC.^115^ Pain control is another critical factor, with multimodal, non-opioid protocols such as Enhanced Recovery After Surgery (ERAS) improving recovery and long-term QoL by promoting early ambulation and reducing opioid dependence.^116^

## Psychological and Social Impact

 Patients also experience varying degrees of psychological distress, an overlooked yet profound issue arising from cancer diagnosis, treatment side effects, and the emotional burden of the disease’s physical symptoms.^117^

 With anxiety and depression affecting up to 57% of cancer patients, far higher than in the general population, significantly impacting mental well-being, treatment adherence, and overall QoL.^118,119^ To address these challenges, organizations such as the ASCO emphasize mental health evaluation and interventions, including counselling, pharmacotherapy, and psycho-oncology referrals.^114^ Behavioural activation (BA), a promising approach for managing psychological distress, empowers patients to adopt adaptive coping strategies. In a randomized trial involving 139 patients with esophageal and gastric cancers, BA was shown to reduce anxiety and improve self-efficacy. Another study of 153 patients with advanced esophageal and gastric cancer found that BA significantly reduced cancer-related stigma and improved overall QoL, making it a practical intervention that primary care providers can implement without specialized training.^117,118^

 Prehabilitation, combining physical activity, nutritional optimization, and psychological support between diagnosis and treatment, has also proven effective in improving physical fitness, mental health, and surgical outcomes in GEC patients.^120^

## Financial Burden

 Patients with GEC and their caregivers face substantial financial hardship due to the high cost and intensity of treatment, which often leads to poor outcomes.^121^ While newer drugs such as nivolumab-based regimens have extended survival, they significantly increase costs by over $95,000 per patient, with cost-effectiveness ratios exceeding $386,000 per quality-adjusted life year (QALY) compared with chemotherapy alone.^122^ Beyond direct medical expenses, financial toxicity encompasses out-of-pocket costs, income loss, and long-term financial strain, all of which negatively impact mental well-being, QoL, and treatment adherence.^123^ Proactive financial navigation programs have shown promise in mitigating this burden and improving QoL for patients and caregivers by addressing insurance challenges and reducing out-of-pocket expenses.^121^

## Artificial Intelligence (AI) in Gastric and Esophageal Cancers

 AI is transforming healthcare by utilizing advanced algorithms to improve diagnosis, treatment, and clinical decision-making. AI technologies are particularly important in oncology for enhancing cancer interventions and patient care.^124,125,126^ Figure 3 provides an overview of AI applications in GEC diagnostics and treatment planning, highlighting the key areas where AI has a significant impact. For gastric and esophageal cancers, AI addresses the diagnostic challenges posed by the often-asymptomatic nature of these diseases in the early stages, aiding early detection and personalized treatment to improve outcomes.^127^

**Figure 3:**
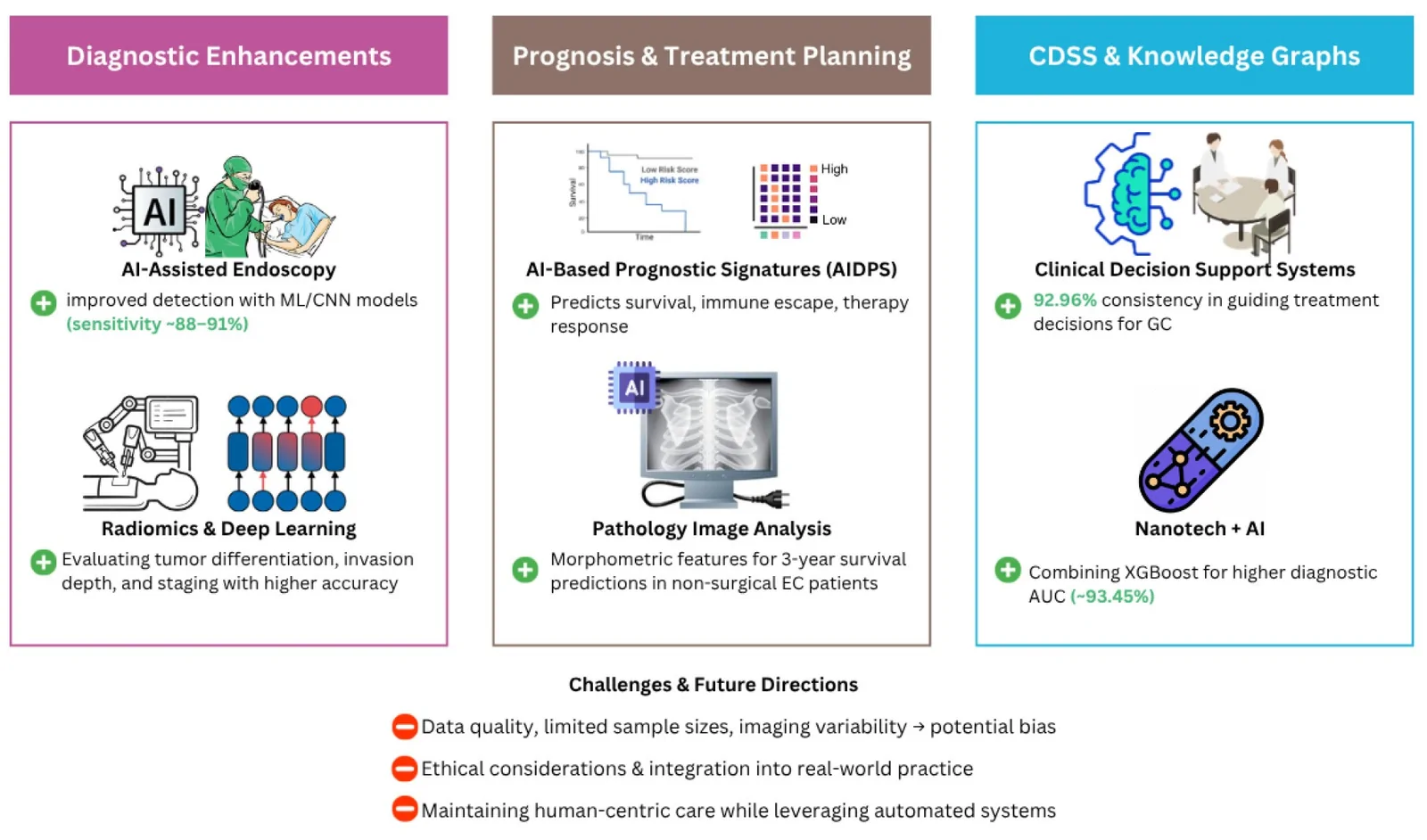
Overview of artificial intelligence applications in gastroesophageal cancer diagnosis and treatment planning

 Endoscopy, the primary diagnostic tool for GC, has operator variability and a 10% miss rate.^128,129^ Machine learning (ML), particularly neural networks (NN), improves diagnostic accuracy by analyzing medical images. Endoscopy-assisted AI technologies trained on diverse imaging datasets can identify lesion boundaries with 88% sensitivity and 89% specificity, thereby reducing operator fatigue and improving patient outcomes.^129^

 The diagnosis of EC is also missed, has high interobserver variability, and highly depends on the endoscopist’s skill and experience.^130^ Various methods have been used to decrease the variability and enhance early detection, including radiomics, which interprets radiological images and analyses images by dividing them into regions of volume of interest and gathering the necessary detailed information. These methods are tailored to specific cancer types and imaging modalities and can assess the degree of tumor differentiation, aiding in efficient diagnosis with more details and less time.^131^ A 2024 study successfully created an AI-based system for the automated recognition of esophageal treatment using ESD phases with high accuracy by evaluating 94 videos of ESD for superficial EC. An NN system was created to identify different phases of the endoscopic procedure. This system was trained using videos labelled and checked by gastrointestinal endoscopists. This AI model achieved an accuracy of 90%, showing potential for clinical use.^132^

 Additionally, a meta-analysis has assessed the use of AI in the endoscopic diagnosis of ESCC in 14 studies with 1590 patients with variable deep learning (DL) models such as convolutional neural networks (CNN), white-light imaging (WLI), and narrow-band imaging (NBI) demonstrated better results when compared with the results of endoscopists alone when diagnosing early stages and assessing the depth of tumor invasion, with a sensitivity of 91.2% and a specificity of 80%, indicating a promising future for AI in the diagnostic field.^133^

 The Artificial Intelligence-based Prognostic Signature (AIDPS) developed in 2024 uses over 100 algorithms to predict prognosis, immune escape mechanisms, and therapy responses, enabling personalized treatments for patients with GC.^134^ Furthermore, in 2024, Li and others proposed a treatment support system using an AI-based Clinical Decision Support System and a knowledge graph with a consistency rate of 92.96 %, possibly guiding doctors in making GC treatment decisions.^135^

 AI tools can also aid in treatment planning. ML models predict radiochemotherapy response and survival rates based on histopathological biomarkers, thereby guiding doctors in developing tailored strategies to improve outcomes. For instance, DL models analyzing morphometric features of stained specimens predicted 3-year survival rates and treatment responses in non-surgical EC patients.^136^

 Nanotechnology, combined with AI, enhances imaging precision and diagnosis of gastrointestinal cancers. ML models integrated with nanotechnology, such as XGBoost, have achieved 93.45% AUC and 93.54% accuracy in analysing gastric images, marking significant progress in early detection.^137^ EC, innovative tools such as bimetallic nanoparticle-catalyzed sensors improve biomarker detection and offer novel diagnostic methods for early-stage cancers.^138^

## Research Gaps and Limitations

 Advancements in precutting EBLR tools are also essential for broadening the application of this minimally invasive technique. There is a critical need for further studies to validate multimodal treatment strategies and rigorously assess the diagnostic accuracy of PET tracers in randomized clinical trials.^139,140^ The biomarker section highlights significant gaps, such as the need to standardize biomarker assays, conduct robust validation studies, and establish the clinical utility of these biomarkers in clinical trials.^141^ Further research should explore tumor heterogeneity and tumor microenvironment to identify reliable biomarkers. Comprehensive studies are also required to identify markers that contribute to therapy resistance, with the aim of developing targeted drug therapies.

 AI in cancer care faces challenges such as limited sample sizes, data quality issues, and imaging variability, which can introduce bias and inequity.^129,142^ Efforts should focus on addressing these limitations, enhancing clinical reliance, and maintaining a humanized approach alongside AI to ensure equitable access and integration into clinical practice.^130,143,144^ Research on QoL improvements and multidisciplinary care approaches requires further investigation into the long-term effects of ICIs on HRQoL.^112^ Studies should also tailor individualized dietary counselling based on different patient characteristics and surgical procedures to improve nutritional status post-surgery.^115^ Psychologically, large-scale, multi-center studies are necessary to validate the global applicability of BA and assess the impact of proactive financial navigation on reducing financial hardship for patients and caregivers.^117,121^

 Lastly, the clinical implementation of ctDNA and PDOs requires standardized protocols, cost reduction, and ethical guidelines to ensure data consistency and security before these technologies can be fully integrated into clinical workflows.^93,95,96^ Overcoming these challenges is essential for leveraging ctDNA and PDOs in personalized medicine and improving treatment outcomes in GEC.

## Conclusion

 This review underscores substantial progress in GEC research, demonstrating that advancements in diagnostic technologies, biomarker use, and targeted therapies are beginning to shift the landscape of patient care. Despite these promising developments, the actual impact on patient survival and QoL remains constrained by the late-stage diagnosis and the complex nature of these cancers. It is imperative that the next phase of research not only focuses on refining these emerging technologies but also ensures their accessibility and integration into global healthcare systems. This approach bridges the gap between innovative research and real-world clinical outcomes, with the aim of delivering tangible improvements in survival rates and patient well-being.
